# Structural and Ultrastructural Morphological Evaluation of Giant Anteater (*Myrmecophaga* *tridactyla*) Prostate Gland

**DOI:** 10.3390/biology10030231

**Published:** 2021-03-17

**Authors:** Fernanda Moura, Letícia Sampaio, Priscila Kobayashi, Renee Laufer-Amorim, João Carlos Ferreira, Tatiane Terumi Negrão Watanabe, Carlos E. Fonseca-Alves

**Affiliations:** 1 Department of Veterinary Surgery and Animal Reproduction, School of Veterinary Medicine and Animal Science, São Paulo State University—UNESP, Botucatu 18618-81, Brazil; fernanda.barthelson@unesp.br (F.M.); leticia.sampaio@unesp.br (L.S.); joao.cp.ferreira@unesp.br (J.C.F.); 2 Department of Veterinary Clinic, School of Veterinary Medicine and Animal Science, São Paulo State University—UNESP, Botucatu 18618-81, Brazil; priscila.e.kobayashi@unesp.br (P.K.); renee.laufer-amorim@unesp.br (R.L.-A.); 3 Department of Population and Health Pathobiology, College of Veterinary Medicine, North Carolina State University, Raleigh, NC 24607, USA; tnegrao@ncsu.edu; 4 Institute of Health Sciences, Paulista University—UNIP, Bauru 17048-290, Brazil

**Keywords:** xenarthra, morphology, immunohistochemistry, reproduction, conservation

## Abstract

**Simple Summary:**

The giant anteater (*Myrmecophaga tridactyla*) is a vulnerable species that lives in South America, extinct in several countries. In the past year, different governmental and non-governmental programs were created for the giant anteater’s conservation. However, little is known regarding the reproductive aspects of this species. Thus, to the best of our knowledge, this is the first description of a morphological and ultrastructural analysis of the prostate gland.

**Abstract:**

The giant anteater (*Myrmecophaga tridactyla*) is a vulnerable species from Central and South America, and is considered possibly extinct in Belize, Guatemala, El Salvador, and Uruguay. Due to the species’ conservation and reproductive importance, this research aimed to characterize the morphology, histochemical, immunohistochemical, and ultrastructural feature of the giant anteater prostate gland. For this, we collected 11 giant anteater prostate glands and performed macroscopic, morphological, histochemical, immunohistochemical, and ultrastructural analysis. Nine prostate glands from an adult subject and two from young subjects were studied. Grossly, the adult giant anteater prostate gland is divided in two distinct zones; the central zones (composed mainly of ducts) and the peripheral zones (of acini formed by secretory cells). The secretory cells showed positive periodic acid–Schiff staining. Furthermore, the immunohistochemical characterization revealed a similar human prostate pattern, with p63 staining basal cells, uroplakin III (UPIII) superficial cells of prostatic urethra, androgen receptor (AR) expressing nucleus of secretory and stromal cells, and prostatic specific antigen (PSA) staining prostatic epithelial cells. Overall, our research provided an in-depth morphological description of the giant anteater’s prostate gland, providing valuable information for futures studies focused on giant anteater conservation.

## 1. Introduction

The giant anteater (*Myrmecophaga tridactyla*), also known as the ant bear, belongs to the Myrmecophagidae family and can only be found in the tropical and subtropical areas of Central and South America [1,2,3]. The giant anteater is considered possibly extinct in Belize, Guatemala, El Salvador, and Uruguay. Overall, it is considered “vulnerable” on the International Union for Conservation of Nature (IUCN, Gland, Switzerland) Red List of Threatened Species [1,2]. Several factors contribute to population reduction and even extinction of the giant anteater, including predatory hunting, forest burnings, and lack of governmental programs for conservation [1,2]. Ongoing intensive growth of agricultural activities has a critical economic role in many countries in South America; however, programs that encourage the awareness and protection of vulnerable species are still scarce [1,2]. Since 2019, Brazil is the fourth country in the world with the higher number of mammals included in the IUCN red list [1,2]. In addition, giant anteaters have slow reproductive cycles in nature, since they present long gestation periods (i.e., one cub born per year), and the type of parental care can also contribute to the species decline [1,2].

Even though the giant anteater is considered a “vulnerable” species, reported studies—regarding the anteater’s reproductive aspects, its association with conservation programs, and development of protocols emphasizing new reproductive biotechnologies for this species—are extremely scarce [4,5,6,7]. Although the number of sexual accessory glands varies for different mammals, the prostate gland is present in all mammal species, and it is considered one of the most important [8,9].

Male dogs and male human beings are the species with the most detailed prostatic studies with complete and broad information, which include full organ description based on macroscopy, histology, and immunohistochemical patterns [8]. Among the immunohistochemical markers used in veterinary medicine, prostatic specific antigen (PSA), p63, uroplakin III (UPIII), and androgen receptor (AR) were already well-described, especially in dogs [9]. To our knowledge, a complete prostatic characterization in the giant anteater is yet to be investigated. Therefore, this research aimed to characterize the prostate gland in the giant anteater by morphological, histochemical, immunohistochemical, and ultrastructural to aid the conservation program to this species in future.

## 2. Materials and Methods

### 2.1. Ethical Approval

Three independent Brazilian committees approved this study: the National Management System for Genetic Heritage and Associated Traditional Knowledge (#A1B0E09), Chico Mendes Institute for Biodiversity Conservation (#65487), and the ethics committee on the use of animals in research (CEUA) at Sao Paulo State University (#0002/2018). Postmortem sampling collection was done at the Department of Veterinary Pathology Service from Sao Paulo State University (UNESP).

### 2.2. Samples and Morphological Analysis

#### 2.2.1. Samples Collection

Tissues were collected from anteaters within 6 h of accidental death from wildlife-vehicle collision. Thirteen free-living giant anteaters were autopsied within six hour intervals after death due to blunt trauma force. Fresh prostate glands were initially macroscopically evaluated based on anatomical measurements and organ weight. For microscopic examination, the prostatic tissue samples and testis were fixed in neutral-buffered 10% formalin for 24 h, embedded in paraffin, and routinely processed for histologic examination in formalin-fixed, paraffin-embedded (FFPE). Additionally, samples were fixed in pentane—1,5-dial glutaraldehyde for 24 h at 4 °C for scanning electron microscopy. The individual subjects’ information, including body weight and prostate measurement, are shown in Table 1.

All of the histological sections were stained with hematoxylin and eosin (H&E), Masson’s trichrome stain, and Periodic acid-Schiff stain (PAS). Morphological analysis of the giant anteater’s prostatic gland followed the standards established by Rossi et al. (2013) [7].

#### 2.2.2. Scanning Electron Microscopy

The prostate sample selected for scanning electron microscopy analysis (MEV-EDX, FEI Company, Quanta 200 e EDX da Oxford, 51-XMX1119) was previously fixed in pentane—1,5-dial glutaraldehyde for 24 h at 4 °C.

#### 2.2.3. Immunohistochemistry

The tissue sections obtained from the FFPE blocks were placed on charged slides (Starfrost, Knittel, Bielefeld, Germany). For the antigen retrieval, the slides were incubated in a citrate solution (pH 6.0) in a pressure cooker (Pascal, Dako, Carpinteria, CA, USA) for 45 min. Endogenous peroxidase was blocked by hydrogen peroxide 8% diluted in methyl alcohol for 15 min and treated with skim milk 8%, at room temperature. The sections were incubated overnight at 4 °C with primary antibodies anti-AR (1:50; clone ab77557; Abcam), anti-PSA (1:1500; polyclonal; Bioss), anti-P63 (1:100; clone 4A4; Dako, Carpinteria, CA, USA), and anti-uroplakin III (UPIII; 1: 50; clone AU1, Researches technologies). A polymer detection system (Envision, Dako, Carpinteria, CA, USA) was applied as a secondary antibody, and immunoreactive cells were visualized by colorimetric detection (3,3ʹ-diaminobenzidine). The sections were counterstained with Harris hematoxylin (Dinamica-Diadema, Diadema, Brazil). A canine prostate was used as a positive control for all antibodies. Negative controls were applied to one section of canine prostate.

The sexual maturity estimation of studied free-living giant anteaters was based on the criteria reported by Smith (2007), Baugher (2004), and Jerex and Haloy (2003), resulting into two studied groups: adult vs. young. In brief, young animals were between 10 months and two years of age, adult animals were at least 30 kg and sexually mature. The testicular maturity was determined by the presence of spermatogenesis in FFPE testis tissue samples.

#### 2.2.4. Histochemistry

The immunostaining evaluation for all antibodies was classified as negative or positive expression in the membrane, cytoplasm, or nuclear immunolabeling.

## 3. Results

### 3.1. Animals

Thirteen free-living giant anteaters were referred to the Wild Animal Service at the Veterinary Teaching Hospital at São Paulo State University after a history of blunt trauma secondary to wildlife-vehicle collisions between January 2017 and December 2019. After postmortem examinations, 11 prostatic samples from adults (>2-year-old) and two prostatic samples from young (<2-year-old) were macroscopically and microscopically assessed. The age category was based on the total body weight and crump-to-rump length.

#### Structural and Ultrastructural Evaluation

- Macroscopic analysis, microscopic assessment and histochemical evaluation;
- Scanning electron microscopy;
- Immunohistochemistry.

**Figure 1:**
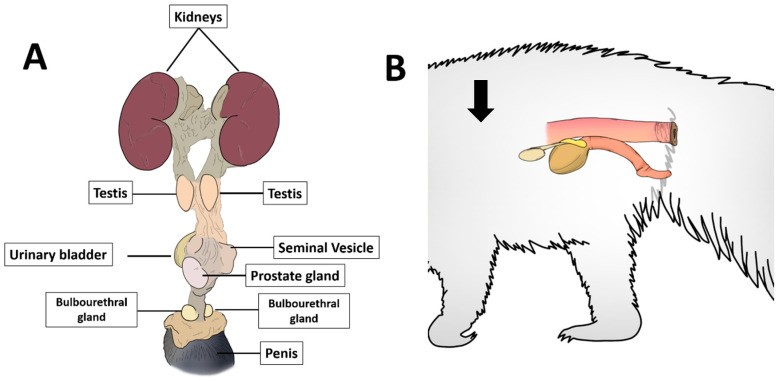
Schematic representation of anatomic localization of giant anteater prostate gland. (**A**): representation of the prostate gland dorsal do the urinary bladder (and ventral to the rectum. (**B**): schematic representation of the giant anteater urogenital tract showing the testicles cranial to the urinary bladder, in which it is possible to observe dorsally the prostate, all lying within the abdominal cavity (arrow).

The giant anteater encapsulated prostate is located dorsally to the urinary bladder and ventrally to the rectum (Figure 1).

**Figure 2:**
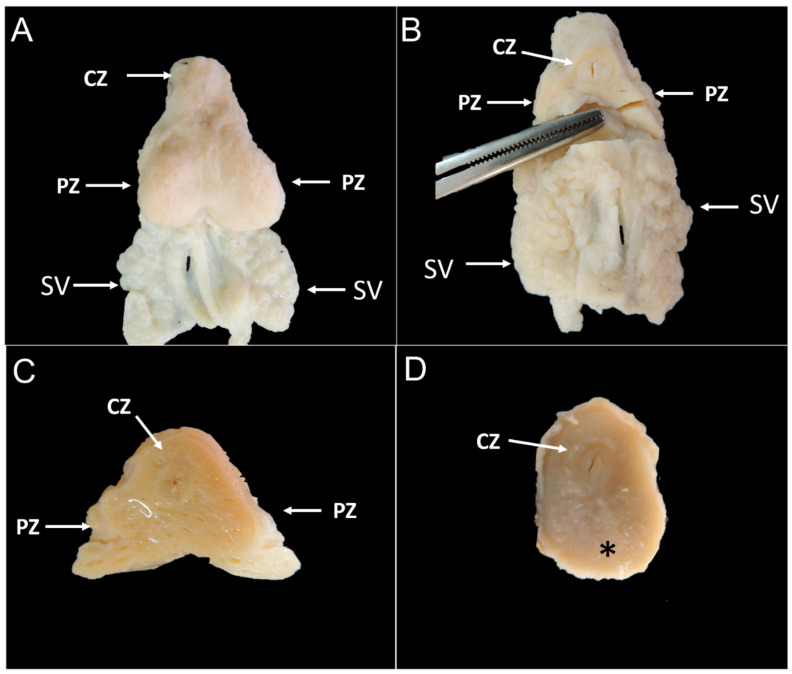
Gross image of adult (**A**–**C**) and young (**D**) giant anteater prostate glands. (**A**): it is possible to observe a bilobulated gland with a fibrous capsule. (**B**): transverse section showing a central zone surrounding the prostatic urethra and the two peripheral lobes (peripheral zones). (**C**): macroscopic appearance of the giant anteater prostate gland at transverse section, with the peripheral zones characterized by the presence of several cavities filled with a gelatinous material, (arrow) (**D**): transverse section showing a central prostatic urethra surrounded by a central zone and no clear definition of the peripheral zones (asterisk). CZ: central zone; PZ: peripheral zone; SV: seminal vesicle.

**Figure 3:**
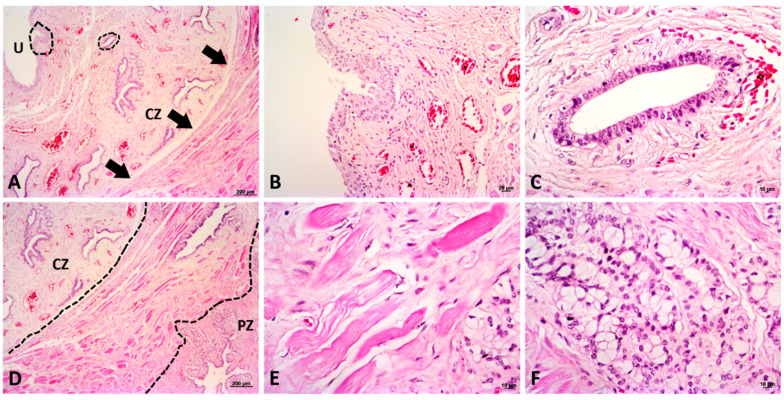
Histological representation of the different prostatic zones. (**A**): low magnification (5×) of central zone of the prostate gland, showing the prostatic urethra (U) and a high density of blood vessels and ducts. The lined circles represent a higher magnification of the prostatic structures showed in figures B and C. It is also possible to observe a muscle-rich stroma surrounding the central zone (arrows). (**B**): a high magnification (20×) of the prostatic urethra. (**C**): a higher magnification of a prostatic duct of the central zone (CZ). (**D**): a low magnification (5×) of the division of both zones: central zone (CZ) and peripheral zone (PZ). The muscle-rich stroma surrounds the prostatic urethra (in the middle of the dotted lines), dividing the central zone (enriched by ducts) and peripheral zone (enriched by luminal glands). (**E**): a higher magnification (40×) of the stroma dividing both zones. This region is composed by a muscle-rich stroma. (**F**): luminal glands from the peripheral zone (in the figure D, showed in the lower left side). Hematoxylin and eosin staining.

Based on the macroscopic findings of the prostate gland, we proposed two different anatomical zones: central and peripheral zones. The central zone is located very centrally in a transverse section and represents the prostatic parenchyma that surrounds the urethra (Figure 2 and Figure 3). The peripheral zones are located at the periphery of the gland and have two grossly distinct lobes without any bridging communication between each other: right and left lobes. Both lobes are replete with gelatinous transparent fluid within multiple cavity foci (Figure 2). Interestingly, the two young subjects had no clear distinction between the peripheral prostatic lobes (Figure 2).

**Figure 4:**
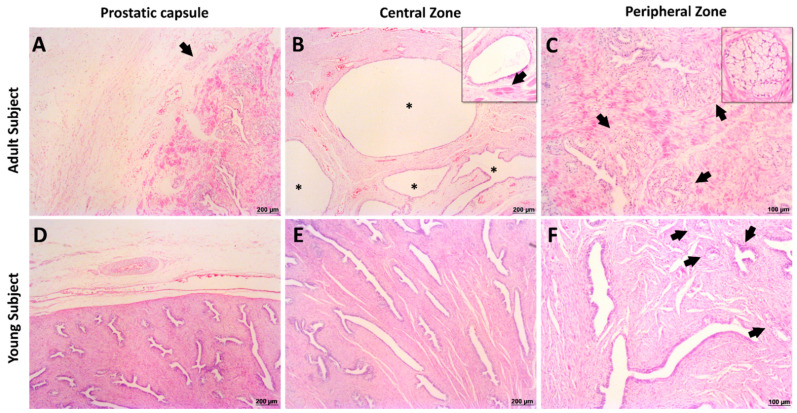
Histological evaluation of giant anteater prostate gland. (**A**): peripheral region of the prostate gland capsule of an adult subject presenting the peripheral nerves (arrow) and blood vessels. (**B**): central zone of the adult giant anteater, presenting prostatic ducts (insert) with large lumen (asterisk). (**C**): peripheral zone showing a central duct surrounded by secretory cells (insert). It is observed a high density of luminal secretory cells (arrows). (**D**): peripheral region of a young giant anteater capsule showing large caliber blood vessels. (**E**): central zone of a young giant anteater enriched by ducts with columnar epithelium. (**F**): peripheral zone of a young giant anteater prostate gland present high caliber ducts and small cluster with secretory cells (arrows). Hematoxylin and eosin staining.

**Figure 5:**
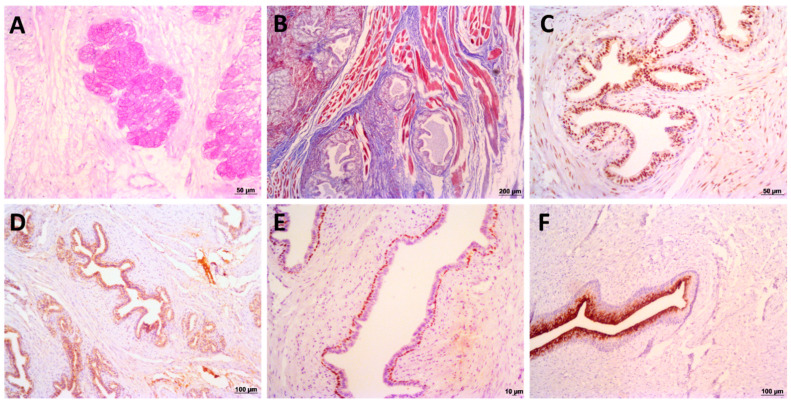
Histochemical and Immunohistochemical analysis of giant anteater prostate gland. (**A**): luminal cells from peripheral zone presenting PAS-rich secretory cells. (**B**): Masson’s trichrome stain, presenting muscle fibers staining in red and collagen fibers stained in blue. It is possible to note a fibromuscular stroma in peripheral zone. (**C**): androgen receptor (AR) nuclear immunoexpression in prostatic epithelial cells. (**D**): prostatic specific antigen (PSA) staining in prostatic epithelial cells. (**E**): p63 positive expression by basal prostatic cells. (**F**): uroplakin III immunoexpression by urothelial superficial cells from prostatic urethra.

Microscopically, the prostatic capsule is composed of loose vascular connective tissue some arteries, veins, nervous ganglions and some muscles bundles (Figure 4). Thus, the histological findings provided strong support for the giant anteater prostate gland’s proposed division into these two distinct zones. In the central zone, it was visualized fibromuscular stroma filled with the prostatic ductal epithelial cells, and the prostatic urethra, characterized by transitional epithelium (Figure 4). In Masson trichrome stain, it was possible to identify the collagen and muscle bundles, staining in blue and red, respectively. The ducts epithelial cells were also positive for Periodic acid-Schiff (PAS) stain (Figure 5). In the peripheral zone, there was a stromal fibrous connective tissue with linear fibroblast, arterioles, and venules adjacent to the glycogen-rich acini, which are composed of a pseudostratified columnar epithelium. We observed that the collagen fibers, blue colored with the Masson trichrome stain, and the glycogen-rich epithelial cells were positive for PAS stains (Figure 5). The peripheral zone of the young subjects had 50% fewer numbers of acini when compared to the adult (Figure 4). Furthermore, the central zone in the young category did not shown any macroscopic differences when compared to the adult; however, there was lower numbers of prostatic ducts histologically (Figure 4).

**Figure 6:**
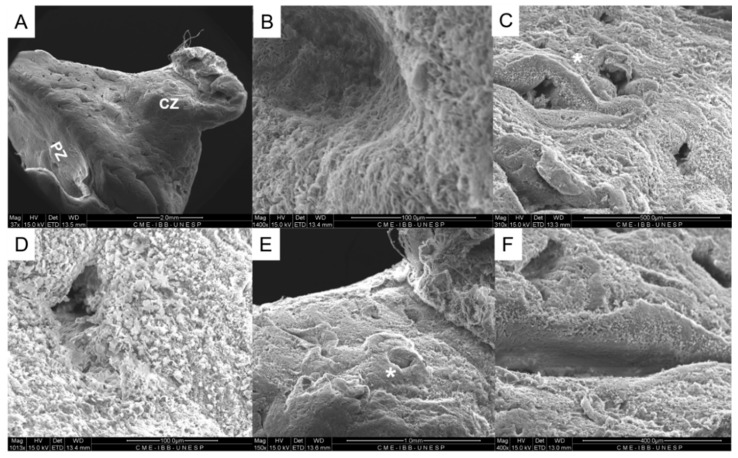
Scanning electronic microscopy. Ultrastructural lower magnification distinguishing the central and peripherals zones (ZC to the central zone and ZP to the peripheral zone, in picture (**A**). It is possible to visualize the capsule (**B**), the peripheral zone with the acinus (asterisk) (**C**), whose epithelium can be observed in higher magnification (**D**), the urethra prostatic in the central zone (asterisk) (**E**), surrounded by the prostatic ducts (**F**).

In the scanning electron microscopy, it was possible to observe a capsule surrounding the prostate gland, the central urethral area and two prostatic lobes regions composed of collagen fibers (Figure 6). In the peripheral lobes, it was observed acinar structures, and their epithelium, whereas in the central zone, it was possible to visualize the prostatic ducts around the urethra (Figure 6).

Androgen receptor (AR) showed intense positive nuclear staining in all prostatic epithelial cells (ducts and acini) and low/absent nuclear expression in the prostatic stromal cells in this species (Figure 4). Furthermore we also observed Immunolabeling anti-PSA was moderate cytoplasmic in all acinar and ductal cells (Figure 4). We observed moderate nuclear P63 expression forming a continuous basal cell layer in the duct and acini of giant the anteater prostate gland (Figure 4). Furthermore, membranous uroplakin III strong positive expression on superficial urothelial cells (umbrella cells) in the prostatic urothelial epithelium was also seen (Figure 4).

## 4. Discussion

Limited information about the giant anteater reproductive tract challenges conservation efforts for this species. A previous study demonstrated the sequential ejaculate fractions in giant anteaters submitted to electroejaculation, which is composed of two different sequential fractions [10]. A study on the lesser anteater (*Tamandua tetradactyla*), describing the gross histopathological morphology of the male genital tract, has also been reported. However, to the best of our knowledge, no previous studies describing the structural and ultrastructural morphology of the giant anteater prostate gland has been published to this date.

The macroscopic and morphological findings of this study strongly support our proposed division and classification of the giant anteater prostate gland in two different zones: central and peripheral. On the other hand, the human prostate is divided into three zones: central, transitional, and peripheral zones [8]. The criterion supporting the prostate gland division into central and peripheral zones is the histologic differences between the zone’s stroma and parenchyma, including the number of vessels, percentage of ducts and acini, and the presence of striated muscle in the central zone. To our knowledge, this is a novel prostatic classification in giant anteaters and can be considered one of the differences found between this specie and its Xenarthra parent, the lesser anteater [7].

The immunohistochemistry characterization of the great anteater prostate gland allowed us to compare our findings with human and canine [11]. Human, canine, and giant anteater prostate present some similarities, including the positive expression for AR, PSA, P63, and UPIII. Interestingly, the giant anteater prostate gland has a continuous basal cell layer similarly seen in humans; however, it differs from dogs, rats, and mice due its discontinuous basal cell layer [8,12,13].

Furthermore, the androgen receptors and the PSA markers showed a positive expression in luminal prostatic cells. On the other hand, the transitional cells from the prostatic urethra showed immunoreaction anti UPIII. Thus, the expression pattern of the prostate markers is typical among giant anteater humans and dogs [8,9].

It is important to emphasize that we observed that the giant anteater’s prostatic stroma has striated muscle bundles, in Masson’s trichrome stain. Usually, the prostate gland of different mammals presents only smooth muscle fiber. For example, the prostatic stroma of human prostate is mostly smooth muscle, while in the mouse and rat, the prostatic ducts are surrounded by thin smooth muscle bundles [12,13]. The presence of striated muscle fibers suggests that the giant anteater prostate gland may have a voluntary muscle contraction control system. Another histochemical technique used, the PAS stain, allowed us to visualize the glycogen presents in the prostatic acini and ducts.

Finally, it is essential to emphasize that the giant anteater is a vulnerable species, which nowadays suffers constant losses in its population. The main contributing reason for this decrease is due to increasing human presence in their habitats and their slow reproductive cycle [1,3,4]. The characteristics of the giant anteater prostate study, such as morphological variations between young and adult subjects’ characterization of the zones, and, especially, the presence of striated musculature in the central zone, raise a hypothesis for possible autonomous control of the prostatic fluid ejaculate, as observed in human and canine prostates [14]. Additionally, it is possible to speculate the importance of this findings involving the participation of this gland in the giant anteater’s fertility. Therefore, further studies involving the description of the prostatic innervation and the other accessory glands are welcomed to develop conservation and breeding programs.

## 5. Conclusions

This study provides the first morphological and immunohistochemical description of the giant anteater prostate gland. Overall, our results suggests that the giant anteater prostate gland may divide into two different zones—a central zone enriched by ducts and the peripheral zone, enriched by luminal secretory cells.

## Figures and Tables

**Table 1:** Giant anteater autopsied information.

Identification	Age	Weight (Kg)	Central Zone	Peripheral Zone	
				Left Zone Size (cm)	Right Zone Size (cm)
**Subject 1**	Young	19	3.1 × 3.5 × 1.9	3.5 × 4.1×3.1 *	NA
**Subject 2**	Young	22	3.9 × 3.2 × 2.5	3.9 × 4.0 × 2.9 *	NA
**Subject 3**	Adult	38	5.6 × 6.1 × 4.3	7.1 × 3.7 × 5.6	7.2 × 3.5 × 5.4
**Subject 4**	Adult	40	6.1 × 5.4 × 4.9	6.9 × 4.1 × 5.4	6.7 × 4.4 × 5.6
**Subject 5**	Adult	37	5.6 × 5.5 × 4 × 2	7.3 × 4.7 × 5.1	7.1 × 4.9 × 5.1
**Subject 6**	Adult	35	5.4 × 4.9 × 4.1	5.9 × 2.7 × 4.9	6.2 × 3.1 × 4.8
**Subject 7**	Adult	34	5.8 × 5.1 × 3.9	6.1 × 4.3 × 4.8	6.3 × 4.2 × 4.7
**Subject 8**	Adult	37	5.6 × 4.9 × 4.1	6.5 × 4.1 × 5.0	6.3 × 4.1 × 5.1
**Subject 9**	Adult	38	5.9 × 3.9 × 3.8	6.8 × 4.0 × 4.9	6.9 × 4.2 × 4.7
**Subject 10**	Adult	39	6.1 × 5.7 × 4.5	7.3 × 4.2 × 5.3	7.1 × 4.4 × 5.2
**Subject 11**	Adult	33	5.3 × 5.1 × 5.2	6.5 × 2.9 × 5.0	6.6 × 2.9 × 4.8
**Subject 12**	Adult	38	5.4 × 4.9 × 4.7	6.9 × 4.7 × 4.2	7.0 × 4.7 × 4.5
**Subject 13**	Adult	39	5.7 × 5.1 × 4.9	7.1 × 3.3 × 5.0	7.1 × 3.3 × 5.0

* Size in centimeters.

## Data Availability

Not applicable.
